# Supplementary material for: Asymmetries around the visual field: From retina to cortex to behavior
Source: PLoS Comput Biol. 2022 Jan 10;18(1):e1009771. doi: 10.1371/journal.pcbi.1009771 (PMC8782511; doi:10.1371/journal.pcbi.1009771)
Supplement: Attachment — Submitted filename: ResponseToReviewers_PCOMPBIOL-D-20-01908_v2021124.pdf [file pcbi.1009771.s005.pdf]

Reviewer #1

Title: Radial asymmetries around the visual field: From retina to cortex to behavior

Overall Impression
In this manuscript, Kupers, Benson, Carrasco and Winawer employ the ISETbio computational framework coupled with a simplified modeling of the midget RGC mosaic to examine how retinal image sampling by the cones and midget RGCs and spatial image filtering by mRGCs limit psychophysical performance in a forced choice orientation discrimination task. This approach represents a continuation of their earlier work in which they showed that the asymmetries in cone mosaic and optics account for only 10% of the behavioral results. The present extension leads the authors to conclude that mRGC processing further contributes to the radial performance differences, but that still, this is far from fully accounting for behaviour, and that therefore, cortical processing (such as radial asymmetries in the V1-V2 CMF) must be responsible for much of these differences. The paper is a nice extension of their previous work and I only have a few issues that need to be addressed, as well as a few minor issues/suggestions.

We thank the reviewer for their support and valuable feedback. We addressed the comments point by point below. Doing so has strengthened our ms.

Issues to address:
R1 (1) Given the inability of cone density to explain differences in performance, the authors sought to examine the effect of spatial filtering by mRGCs. However, there is a number of neural processing stages between cone excitations and spatial pooling by mRGCs. One such stage is phototransduction, in which cone excitations are converted in a nonlinear way to cone outer segment photocurrent. This process is modeled in ISETBio, and it has been shown to have a significant effect on performance (see Cottaris, Wandell, Rieke and Brainard (2020) "A computational observer model of spatial contrast sensitivity: Effects of photocurrent encoding, fixational eye movements, and inference engine", JoV, 20(7).) The authors should elaborate on the effect that this processing stage might have on the radial asymmetry of performance. For example, we know that photocurrent gain is down-regulated as the rate of cone excitation increases, and that the rate of cone excitation increases as the cone aperture increases. Since cone density varies asymmetrically with angle, so does cone aperture and therefore the non-linear regulation of photocurrent. It would be interesting to see what radial asymmetries are predicted when assessing performance at the photocurrent level. The rate of cone excitation also affects the temporal dynamics of photocurrent and photocurrent noise (see Angueyra and Rieke (2013) "Origin and effect of phototransduction noise in primate cone photoreceptors". Nature Neuroscience. 16: 1692-700). What are the effects of these factors on performance in this task? The authors could conduct an assessment of some of these factors within existing functionality in ISETBio, or at the very least discuss how these factors might contribute towards the observed radially asymmetric performance.

Response: We agree that there are, of course, many steps between isomerization in the cones and outputs of the RGCs. Our rationale for focusing on the RGC spatial properties is the following: A thorough simulation and assessment of all of the stages between isomerization and RGC output is

beyond the scope of this project (and in fact, probably beyond the capability of the field as a whole). We chose to assess the effects of spatial filtering in the RGCs because the RGCs are known to have properties that vary with polar angle, and documenting those properties was a major focus of the paper (**Fig 1 and 2**). It was natural to then assess the impact of those properties.

However, the Reviewer makes a point we had not considered, namely that the SNR of the photocurrent might be dependent on aperture size (and hence cell density). We updated our observer model incorporating conversion from isomerization to photocurrent as implemented in ISETBIO, and found that indeed the Reviewer's suspicion panned out exactly: the model performance incorporating photocurrent showed a sharper fall-off with cell density (due to aperture size) than the original model we had used without the photocurrent. This resulted in a horizontal-vertical asymmetry of ~18% predicted by our full model (up to RGCs), rather than ~5% predicted by a model up to cone absorptions. In particular, one can see in the plots of contrast threshold vs cone density that for the model with photocurrents (**Fig 5B**), there is a kink in the curve at lower densities, where performance gets sharply worse. The performance falls off more gradually without the photocurrent (**Fig 5A**). Nonetheless, the 18% asymmetry is still much lower than the asymmetry observed in human observers (~40%). Moreover, incorporating the photocurrent model does not explain any of the upper-lower meridian asymmetry because, unlike mRGC distribution, cortex, and behavior, there is no lower meridian advantage in cone density.

These simulations were informative to us and are in line with the Reviewer's predictions and led to a major improvement in the paper.

Summary: We have added the cone photocurrent simulation results to new **Fig 5-7**, and refer to these results in Results section [starting **p. 16**], and expanded the Methods section with the implementation of the photocurrent simulation [**pp. 33-34**].

R1 (2) The authors mention that their results (for example, that contrast sensitivity at the mRGC layer is higher than at the cone layer) depend on the efficiency of learning of the computational observer. This is a little worrysome. Perhaps a higher level of radial asymmetry would be revealed if the classifier was most sensitive. Or not. The authors could try to examine this by improving the power of the SVM classifier. One way to do this is to apply a stimulus - matched pooling mechanism between the raw responses and the SVM. This pooling would add information about the stimulus, thereby increasing the classifier's learning efficiency, while still preserving radial asymmetries in cell densities. The pooling weights could be derived from the noise-free responses under the no-fixational eye movement condition.

Response: We agree that efficiency of learning of the computational observer model was not meant to be the point of the study, and we do not want this to confound our results. We decided to greatly increase the number of trials in our simulated experiments, to incorporate noise in the conversion of photon absorptions to photocurrent and in the mRGC outputs, and to simplify our simulations by removing fixational eye movements and stimulus uncertainty. We think the results are much clearer and more convincing now, and we no longer find a pattern whereby performance gets better at later stages of the model.

The greater number of trials and dimensionality reduction from temporal averaging reduce the burden of learning for the SVM and make the results more robust. We now find clear and independent effects of cone density and mRGC density on performance, and we find that with each stage of processing,

from photon absorptions to photocurrent to mRGC outputs, the overall model performance declines (as expected) and shows a pattern that gets increasingly closer to human behavior (**Fig 7**).

The expanded Methods sections can be found from **p. 32** onward, with the implementation of photocurrent on [**pp. 33-34**], increased number of trials on [**p. 33 and p. 36**], and noise in mRGC outputs starting from [**p. 35**, last paragraph].

R1 (3) The authors used a patch of mRGCs that had the same field of view as their cone mosaic. This means that mRGCs near the border of the cone mosaic patch had partial driving of their surround subregions. Since mRGC surrounds are quite large, a significant fraction of the modeled mRGCs might suffer from this partial surround coverage. I wonder what the effect of this artifact may be on their results. The authors should provide some discussion of this or perhaps demonstrate that this is not of significant concern.

Response: The Reviewer is correct that the mRGC surrounds can be quite large, especially for the simulated cone mosaics with low cell density. This partial coverage of RGCs introduced zero padding of the cone array in our convolution step (also pointed out by **Reviewer 2, query 3**) and caused border artifacts in our simulated mRGC responses. As filtering a smaller section of cone array is not a feasible alternative for those patches with low density, we have now avoided this artifact by padding cone photocurrent with the mean values from the retinal patch. This changed the results numerically but only slightly, with no change in the overall patterns. See update to **Fig 5-7**, **Supplementary Fig 3**, and to Methods [**pp. 35-36**].

Minor issues/suggestions/corrections:

R1 (4) Did the authors vary the applied optics with polar angle as they did in their earlier paper?

Response: We did not vary the applied optics in this paper. We have clarified this in the Results [**p.12**, in Retinal irradiance section] and Methods [**p. 33** under the section Computational observer model].

R1 (5) Introduction:
Line 10: "better along the lower than upper vertical meridian". Please specify that you refer to visual field meridians.

Response: We have clarified this [**p.4**, second paragraph].

R1 (6) Page 7, Second paragraph:
"The radial asymmetries in cone density are larger in the mRGC distribution".
This should be "The radial asymmetries in density".

Response: We have fixed this [**p.8**, last paragraph].

R1 (7) Page 9, Line 7:
(Fig2A, middle panel), should be (Fig 2B, left panel).

Response: We have fixed this [**p.10**, first paragraph].

R1 (8) Figure 2 caption. Typo.
"These radial asymmetries are not depended on the type".

Response: We have fixed this [**p. 9**].

R1 (9) Figure 2. Suggestion.
To better visualize the differences in radial asymmetries, it might be a good idea to also plot ratios of convergence ratios (horizontal vs. superior and horizontal vs. inferior) across eccentricity.

Response: We have done so and added this plot to **Supplemental Fig 2**.

R1 (10) Figure 7A. Y-axis label
Why is there a '%' in contrast sensitivity on the y-axis label? Same for text in Figure 7 caption and for text in page 20, third paragraph ('with contrast sensitivity about 40%').

Response: This is a typo. We have fixed this in **Fig 7,** its caption, and paragraph of the corresponding Results section [**pp. 19-20**].

Reviewer #2

In “Radial asymmetries around the visual field: From retina to cortex to behavior,” the authors report a suite of insights into neural and perceptual asymmetries along the horizontal and vertical meridians of the retina. This is a thoughtful, detailed, creative, and extremely well written treatment of this issues, which makes several notable advances on prior work on this topic. The author’s recent prior work (also published in PLOS CB) focused on meridional asymmetries in optics and cone photoreceptor density; in the current report there are two key new insights. First, the authors systematically leverage anatomical and functional data to show that these asymmetries are present and amplified at the levels of retinal ganglion cell density and cortical magnification factor in early visual cortex. Second, they show that the spatial filtering properties of midget retinal ganglion cells, combined with asymmetric density long the meridians, augment the predicted asymmetries of observer models, but still do not account for the quite large differences measured in perceptual experiments. My comments that follow are all minor and mostly suggestions for ways to improve the clarity and completeness of the report.

We thank the reviewer for their support and valuable feedback. We addressed all comments in the new manuscript and refer to the page and paragraphs below. Doing so has strengthened our ms.

R2 (1) Intro/Terminology: The authors use the term “radial asymmetries” to refer to variations as a function of polar angle (pg 3). I found this confusing. To me, these variations would be more accurately referred to as “polar angle asymmetries” or “meridional asymmetries.” The term, “radial asymmetries” implies that the variations appear as a function of radius. In pg 23, the authors even use the phrase “radial asymmetries” and “polar angle asymmetries” to refer to the same phenomenon of interest in two consecutive sentences. It doesn’t seem appropriate that both of these terms be used to refer to the same thing, and I think the latter is more clear.

Response: We agree. Some prior papers used “radial asymmetries” in this way (e.g., “Radial asymmetries in population receptive field size and cortical magnification factor in early visual cortex” by Silva et al. 2018) But we agree that is confusing. We now use “polar angle asymmetries” consistently throughout the manuscript and changed the title to “Asymmetries around the visual field: From retina to cortex to behavior.”

Results:
R2 (2) -Pg. 6 (Fig 1): I didn’t understand why the mRGC : V1 CMF ratio decreases again after 20 degrees. Is this expected? It would be helpful if the authors addressed this feature of the data.

Response: We have addressed this issue [**p.7** last paragraph, continuing on p.8].

R2 (3) -Pg 8 (Fig 2): The CMF data derived from the HCP appear to have quite different mRGC : V1 CMF ratios than the ratios derived when the Horton & Hoyt formula is used (Fig 2B). Some of this difference might be because the HCP data are plotted only for the meridians. Would it be possible to add average lines for the HCP CMF data to Fig 2 panels A and B, for a more direct comparison to the formula overall? I also think the inclusion of V2 in the CMF estimates for the vertical meridian, but not the horizontal, warrants further scrutiny. It seems fair to compare superior and inferior in this regard, but is it really fair to compare horizontal to vertical with these data? In addition to the stated

assumption that “V2 is approximately the same size as V1” (pg 29), do other assumptions need to be made about the relative CMF and receptive field sizes in V2 and V1?

Response: We have plotted the average of the HCP CMF data as an additional line in **Fig 2A and B** [**p. 9**]. While the initial surface area data for upper and lower visual field wedge ROIs are partially in V2, we correct the surface area to exclude V2 in our CMF estimates. We revised and extended this Methods section to clarify the CMF computation [**p. 31,** second paragraph starting with “The initial ROIs used for the upper…”] and changed “V1-V2 CMF” to “V1 CMF” throughout the manuscript to avoid confusion.

R2 (4) -Pg. 12 (Fig 3): I understand that a spiking model isn’t used for the RGCs, but it doesn’t seem appropriate to label RGC responses in units of photons/ms. I’d suggest just calling these arbitrary units.

Response: We have fixed this label in **Fig 3** [**p. 13**] and Methods [**p. 35,** starting last paragraph continuing on p. 36].

R2 (5) Pg. 14 (Fig 4): I found panel A confusing, possibly because the locations of the cones were not indicated. Overall, it would be helpful to have a visualization that includes the cone mosaic to show how the mRGCs tile it. Maybe it’s also hard to interpret this figure because the 2:1 ratio is spatially 1:1? (If one linear mRGC represents a pair of ON/OFF cells, pg 31).

Response: We have addressed this issue by adding a new panel C to **Fig 4** illustrating RGCs and their relationship to the cone array in 2D [**p. 15**].

Methods:

R2 (6) -Pg. 32: it should be noted that conv2 uses zero padding. It was unclear to me whether the sub-sampling removed any edge samples that include contributions of this padding. Is that the case? If not, does the padding impact the results?

Response: The reviewer is correct that the convolution assumed zero padding. We re-computed the simulations where we padded array with mean cone photocurrent values rather than 0. This changed the results numerically but only slightly, with no change in the overall patterns. See update to **Fig 6 and 7** [**p. 18 and p.20**], **Supplementary Fig 3**, and to Methods [**p. 35,** second paragraph].

R2 (7) -Pg. 35: I may be missing something, but I didn’t understand why interpolation was used here. Why not train classifiers directly on the appropriate ratios for each meridian at 4.5 deg?

Response: We did not train the classifier directly on the appropriate ratios for two reasons. First, we used a wide range to assess the general effect of mRGC:cone ratio on performance. We think this makes it easier to understand how cone density and mRGC:cone ratio interact and affect performance, especially as the variations across meridians for both cone density and ratios are relatively small. Second, to make our RGC computation tractable and computationally efficient, we use convolution and linear resampling. This choice limits our computation to only use integers of cones for our computation. We have clarified this issue in the Methods [**p. 34**, starting last paragraph, into p. 35]. Additionally, we added contour lines on the 3D surface of **Fig 6B** to show the possible cone densities and mRGC:cone ratios that would predict the polar asymmetries as observed in behavior at 4.5 degrees eccentricity.

General Formatting:

R2 (8) -In Fig 2, the different lines were hard to distinguish when printed. Given that the number of lines is pretty small, I'd suggest applying different line styles and/or saturations

Response: We changed the color scheme in **Fig 2** and have averaged the nasal and temporal retina, representing the horizontal visual field as a single line to reduce clutter [**p. 9**].

R2 (9) -In the revised manuscript, it would be helpful to include line numbers in addition to page numbers

Response: We have done so.
